# Comparative analysis of density histograms and visual scores in incremental and volumetric high-resolution computed tomography of the chest in idiopathic pulmonary fibrosis patients

**DOI:** 10.1007/s11547-020-01307-7

**Published:** 2020-11-30

**Authors:** Gaetano Rea, Marina De Martino, Annalisa Capaccio, Pasquale Dolce, Tullio Valente, Sabrina Castaldo, Angelo Canora, Francesco Lassandro, Marialuisa Bocchino

**Affiliations:** 1 Dipartimento Dei Servizi Diagnostici E Generali, Ospedali dei Colli, Monaldi-Cotugno, Napoli, Italy; 2 Dipartimento Di Medicina Clinica E Chirurgia, Sezione Di Malattie Dell’Apparato Respiratorio, Università Federico II, Napoli, Italy; 3 Dipartimento Di Sanità Pubblica, Università Federico II, Napoli, Italy

**Keywords:** Idiopathic pulmonary fibrosis, Tomography, Densitometry, Visual score

## Abstract

**Background:**

Volumetric high-resolution computed tomography (HRCT) of the chest has recently replaced incremental CT in the diagnostic workup of idiopathic pulmonary fibrosis (IPF). Concomitantly, visual and quantitative scores have been proposed for disease extent assessment to ameliorate disease management.

**Purpose:**

To compare the performance of density histograms (mean lung attenuation, skewness, and kurtosis) and visual scores, along with lung function correlations, in IPF patients submitted to incremental or volumetric thorax HRCT.

**Material and methods:**

Clinical data and CT scans of 89 newly diagnosed and therapy-naive IPF patients were retrospectively evaluated.

**Results:**

Forty-six incremental and 43 volumetric CT scans were reviewed. No differences of density histograms and visual scores estimates were found by comparing two HRCT techniques, with an optimal inter-operator agreement (concordance correlation coefficient >0.90 in all instances). Single-breath diffusing lung capacity for carbon monoxide (DLCO_sb_) was inversely related with the Best score (*r* = −00.416; *p* = 0.014), the Kazerooni fibrosis extent (*r* = −0.481; *p* = 0.004) and the mean lung attenuation (*r* = −0.382; *p* = 0.026), while a positive correlation was observed with skewness (*r* = 0.583; *p* = 0.001) and kurtosis (*r* = 0.543; *p* = 0.001) in the incremental HRCT sub-group. Similarly, in the volumetric CT sub-cohort, DLCO_sb_ was significantly associated with skewness (*r* = 0.581; *p* = 0.007) and kurtosis (*r* = 0.549; *p* = 0.018). Correlations with visual scores were not confirmed. Forced vital capacity significantly related to all density indices independently on HRCT technique.

**Conclusions:**

Density histograms and visual scores similarly perform in incremental and volumetric HRCT. Density quantification displays an optimal reproducibility and proves to be superior to visual scoring as more strongly correlated with lung function.

## Introduction

High-resolution computed tomography (HRCT) of the chest represents the milestone for the identification of differential patterns in the field of diffuse interstitial lung diseases (ILDs). HRCT is pivotal for disease diagnosis, as also suggests the likelihood of alternative possibilities; also, it may help the further characterization of combination phenotypes, such as the co-existence of emphysema or pleural cap thickening, of co-morbidities, and of any likely cause of acute disease progression [1]. ILDs are a leading cause of both disability and early mortality [2]. Chest HRCT ability to distinguish between diseases with poor prognosis, like idiopathic pulmonary fibrosis (IPF) and other ILDs is essential in patient management [3–7]. Despite the availability of studies addressing the application of qualitative and semi-quantitative visual scoring systems for the assessment of disease extent, the results have only partially satisfied the initial expectations because of issues like intra- and inter-reader variations and low reproducibility [8, 9]. Different computer-based quantification methods have been proposed to overcome these limitations to ameliorate objectivity, sensitivity, and repeatability of ILDs detection. Quantification of lung fibrosis can be obtained through the measurement of mean lung attenuation (MLA, average attenuation value of the lung parenchyma), skewness (extent of histogram asymmetry), and kurtosis (how sharply peaked the histogram is) [10]. Analysis of density histograms has been successfully studied both in IPF and in systemic sclerosis (SSc)-related ILD patients with higher sensitivity and reproducibility concerning visual evaluation and good performance even with low radiation dosage [11, 12]. Incremental HRCT has represented the most widely used tool for ILDs detection even in these studies, while the volumetric approach has received much attention only recently [13]. Volumetric HRCT represents a significant advance over the incremental technique as allows sampling of the whole lung volume with no lack of anatomic continuity [14]. Actually, ILD patients are not studied with the same CT technique in all centers, while a variable proportion of them is likely to be shifted from incremental to volumetric CT for disease monitoring.

Given these considerations, this retrospective study aimed to investigate the performance of density histograms along with lung function correlations in IPF patients submitted to incremental or volumetric HRCT of the thorax at the time of first diagnosis. Best and Kazerooni visual scores were also analyzed for comparison [15, 16].

## Materials and methods

### Study population

Eighty-nine newly diagnosed and therapy-naive patients affected by clinically stable IPF, according to the 2011 official criteria [4], referring to our Division between September 2013 and May 2017 were retrospectively included. Overall, 46 incremental and 43 volumetric CT scans along with clinical data were reviewed. A definite UIP pattern was described in 67 patients, and a possible UIP pattern in the remaining 22 cases. Cases with a combined CT pattern of emphysema with an estimated extension as more than 15% were excluded. Demographics and disease staging of the study population are shown in Table 1. Spirometry, lung volumes measurement, and determination of the hemoglobin (Hb)-adjusted single-breath lung diffusing capacity of the carbon monoxide (DLCO_sb_) were performed using a computer-assisted spirometer (Quark PFT, Cosmed) according to international standards [17–19]. The GAP (gender-age-physiology) index was calculated as previously reported [20]. The 6-min walk test (6-MWT) was performed by trained hospital staff according to reference guidelines [21, 22]. Arterial blood gas analysis at rest while the patient was breathing ambient air was also recorded. Systolic arterial pulmonary pressure (sPAP) was estimated through conventional trans-thoracic echocardiography [23]. Lung function parameters are reported in Table 2.

**Table 1:** Demographics and disease staging of the study population

Parameter	Volumetric CT	Incremental CT	*p*
Age (years)	66 ± 7.3	68.6 ± 7.7	0.161
BMI (Kg/m^2^)	28.7 ± 3.7	28.1 ± 4.2	0.550
Gender (M/F)*	23 (79.3)	33 (82.5)	0.738
Smoking history			0.298
Former smokers*	32 (74.1)	36 (78.9)	
Never smokers*	8 (18.5)	10 (21.1)	
Smokers*	3 (7.4)	0 (0)	
GAP stage			0.147
I*	7 (15.6)	13 (29.1)	
II*	7 (15.6)	5 (10.9)	
III*	29 (68.8)	28 (60)	

Data are expressed as mean ± SD; *Absolute value (%)

Abbreviations: BMI, body mass index; GAP, gender age physiology

**Table 2:** Lung function assessment

Parameter	Volumetric CT	Incremental CT	*p*
Arterial pO_2_ at rest at ambient air (mmHg)	69.3 ± 13.6	69 ± 14.2	0.924
SpO_2_	96 [94.8; 97]	95 [93; 96]	0.201
FVC (% pred)	69.4 ± 20.6	67.5 ± 20.7	0.717
FEV_1_ (% pred)	75.7 ± 20.2	73.5 ± 21.8	0.694
FEV_1_/FVC (%)	90.2 ± 13	87.4 ± 11.9	0.387
TLC (% pred)	59.5 ± 19.5	57.6 ± 18.8	0.740
DLCO_sb_ (% pred)	45.1 ± 17	46.4 ± 16.9	0.781
6-MWT distance (m)	370 [274; 484]	455 [399; 496]	0.175
Systolic PAP (mmHg)	33 [28; 42.5]	35 [29; 41.5]	0.819

Data are expressed as mean ± SD or as median [25th; 75th percentile]

Abbreviations: pO_2_ = O_2_, arterial partial pressure; SpO_2_, oxygen saturation; FVC, forced vital capacity; FEV_1_, forced expiratory volume/first second; TLC, total lung capacity; DLCO_sb_, diffusing lung capacity for carbon monoxide single breath; 6MWT, 6-min walking test; PAP, pulmonary artery pressure

### High-resolution computed tomography (HRCT) of the thorax

Incremental or volumetric HRCT examinations were performed on a 64 slice MDCT scan (LightSpeed VCT 64-slice GE, GE Medical System, Milwaukee, WI, USA) in supine position at full inspiration. High resolution technique was applied with a radiation dose range of 1.4–1.6 millisievert (mSv) and 1.9–2.1 mSv, respectively for incremental and volumetric CT. Scanning parameters were 120 kilovolts and 80 mA with the smallest field of view (FOV) related to the patient body habitus for both incremental and volumetric exams and a matrix size of 512 × 512 pixels. Images were reconstructed with a 1.25-mm slice thickness with 10-mm advancement for incremental CT. A 1.25-mm slice thickness using the bone filters was adopted for volumetric images reconstruction. Digital imaging and communications in medicine (DICOM) data for each patient were transferred and stored on PACS (picture archiving and communication system). The lung parenchyma was independently reviewed by two radiologists with more than 15 years of experience in ILD imaging with a window width of 1.600 Hounsfield Units (HU) and level −600 (HU) for analysis.

### CT density histograms and visual scores analysis

**Fig. 1:**
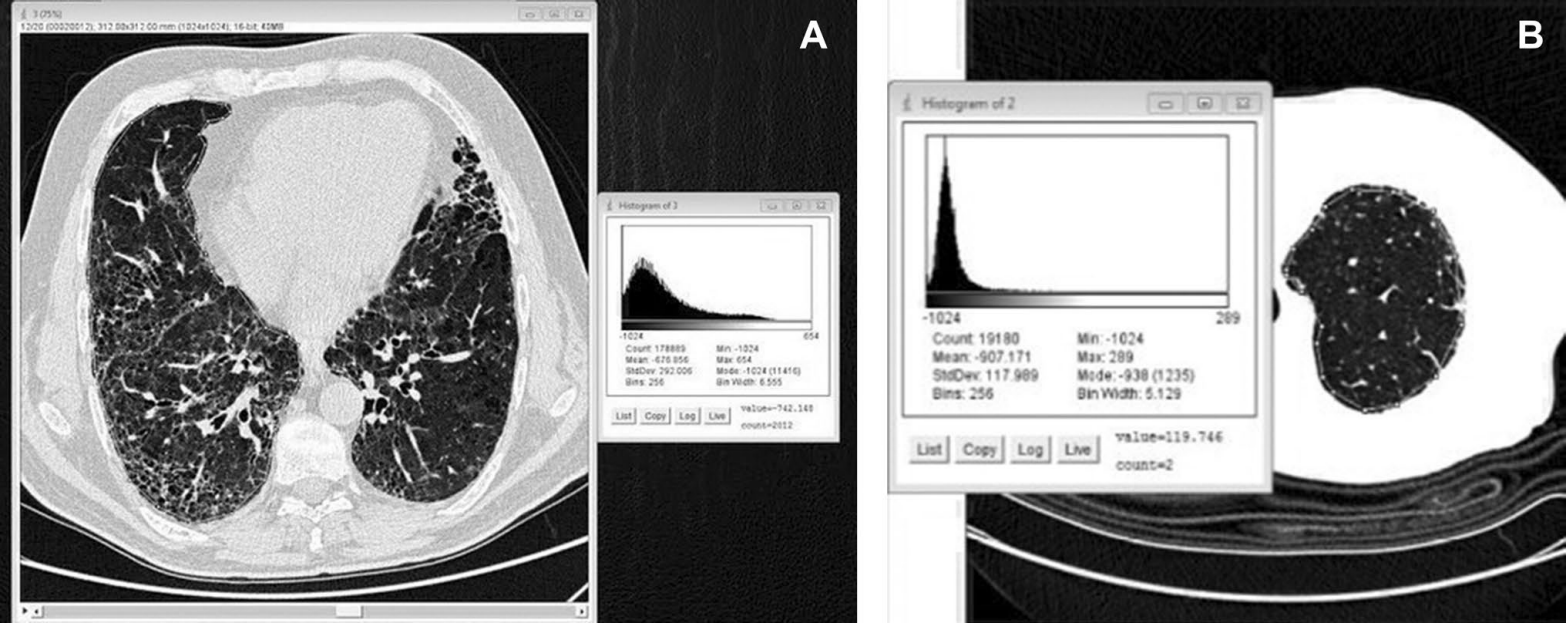
A representative slice segmentation (marked in yellow) of the right lung in a patient affected by advanced idiopathic pulmonary fibrosis and evaluated by means of thin section volumetric CT is shown in panel A. On the right side, results of digital processing analysis of the given slice are reported. After the whole lung sampling was completed, the software automatically generated averaged data from the analysis of all slices of both lungs. The density histogram of the fibrotic lung is both less peaked and less skewed than that of a control normal lung (Gaussian curve sharply shifted to the left with a very narrow and high peak), as reported in panel B for comparison

Histograms of MLA, skewness, and kurtosis were calculated for the entire data set of each patient using a free open-source software for digital image processing (Image J, 1.51 I version, developed by the National Institutes of Health of the USA). This software allowed an automated lung segmentation on axial sections of both incremental and volumetric CT through the preliminary setting of a variable size region of interest (ROI) representative of the lung parenchyma density (ranging from −850 to −910 HU). The ROI was preferentially positioned at the tissue center level of the upper lung lobes free from moderate to severe fibrotic alterations. Such a process was lasting 30–60 s for incremental scans and up to 3 min for volumetric CT. Then, manual segmentation, mainly requiring additional 5–20 min per exam (for incremental and volumetric CT, respectively), was performed slice by slice to rearrange the whole lung surface and ensure the correct inclusion of more peripheral/submantellar advanced fibrotic alterations. Manual segmentation also allowed to exclude anatomical structures, such as the trachea, the bronchi, the main pulmonary arteries, and additional areas of the chest wall that could lead to errors of density quantification [24, 25], as shown in Fig. 1. Each lung was analyzed separately. Finally, MLA, skewness, and kurtosis were then computed slice by slice by digital image processing that automatically allowed the generation of averaged Hounsfield Units (HU) values from all slices. Visual analysis was performed on a remote workstation (Osirix) with the Best and Kazerooni scores, as previously described [15, 16].

### Statistical analysis

Numerical variables were analyzed using mean ± standard deviation (SD). Categorical variables were summarized using absolute frequencies and percentages. Differences between groups were accordingly assessed using either the *t*-test for independent samples and the Chi-square test or the Fisher exact test when appropriate. Correlation analysis was performed with the Spearman test. As previously reported [26], concordance between radiologists was measured using the Concordance Correlation Coefficient (CCC) [27]. CCC values higher than 0.95 were suggestive of substantial agreement. All tests were two-tailed; a *p*-value of 0.05 was considered significant. All statistical analyses were realized with the statistical platform R (The R Formulation for Statistical Computing).

## Results

### Performance of density histograms and visual scores in incremental and volumetric HRCT

Density histograms and visual scores analysis were performed in a retrospective cohort of 89 IPF patients submitted to incremental (*n* = 46) or volumetric (*n* = 43) chest HRCT. As reported in Table 3, estimated measurements of density histograms did not differ when comparing incremental to volumetric CT. Visual score analysis also produced similar results with the two CT techniques. The level of concordance between the two readers was very high, with measurement agreement values ranging from 0.90 to 0.99 in all instances (Table 4).

**Table 3:** Head-to-head comparison of density histograms and visual scoring according to high-resolution CT technique

Parameter	Volumetric CT	Incremental CT	*p*
MLD	−718,201 ± 76,652	−728,451 ± 74,950	0.580
Skewness	1826 ± 619	1783 ± 388	0.738
Kurtosis	4022 ± 3200	3907 ± 2034	0.863
Best score (%)	33.2 ± 12.1	33.8 ± 11.8	0.854
Kazerooni GG score	5 [3.5; 7]	3 [2; 5.8]	0.074
Kazerooni fibrosis score	9 [6; 10.5]	9 [6; 10.8]	0.966

Data are expressed as mean ± SD or as median [25th; 75th percentile]

Abbreviations: MLA, mean lung attenuation; GG, ground glass

**Table 4:** Inter-observer agreement by comparing density histograms and visual scores in volumetric and incremental HRCT

	Volumetric CT	Incremental CT
Density histograms		
MLA	0.98 [0.97–0.99]	0.97 [0.95–0.98]
Skewness	0.96 [0.91–0.98]	0.93 [0.86–0.96]
Kurtosis	0.99 [0.98–0.99]	0.98 [0.97–0.99]
Visual scores		
Best	0.90 [0.81–0.95]	0.97 [0.94–0.98]
Kazerooni (fibrosis score)	0.97 [0.95–0.98]	0.95 [0.90–0.97]
Kazerooni (GG score)	0.97 [0.94–0.98]	0.96 [0.91–0.98]

Data are expressed as Concordance Correlation Coefficients with the corresponding 95% CI’s

Abbreviations: MLA, mean lung attenuation; GG, ground glass

### Density histograms are superior to visual scores as they better correlate with lung function irrespective of the CT technique used

**Fig. 2:**
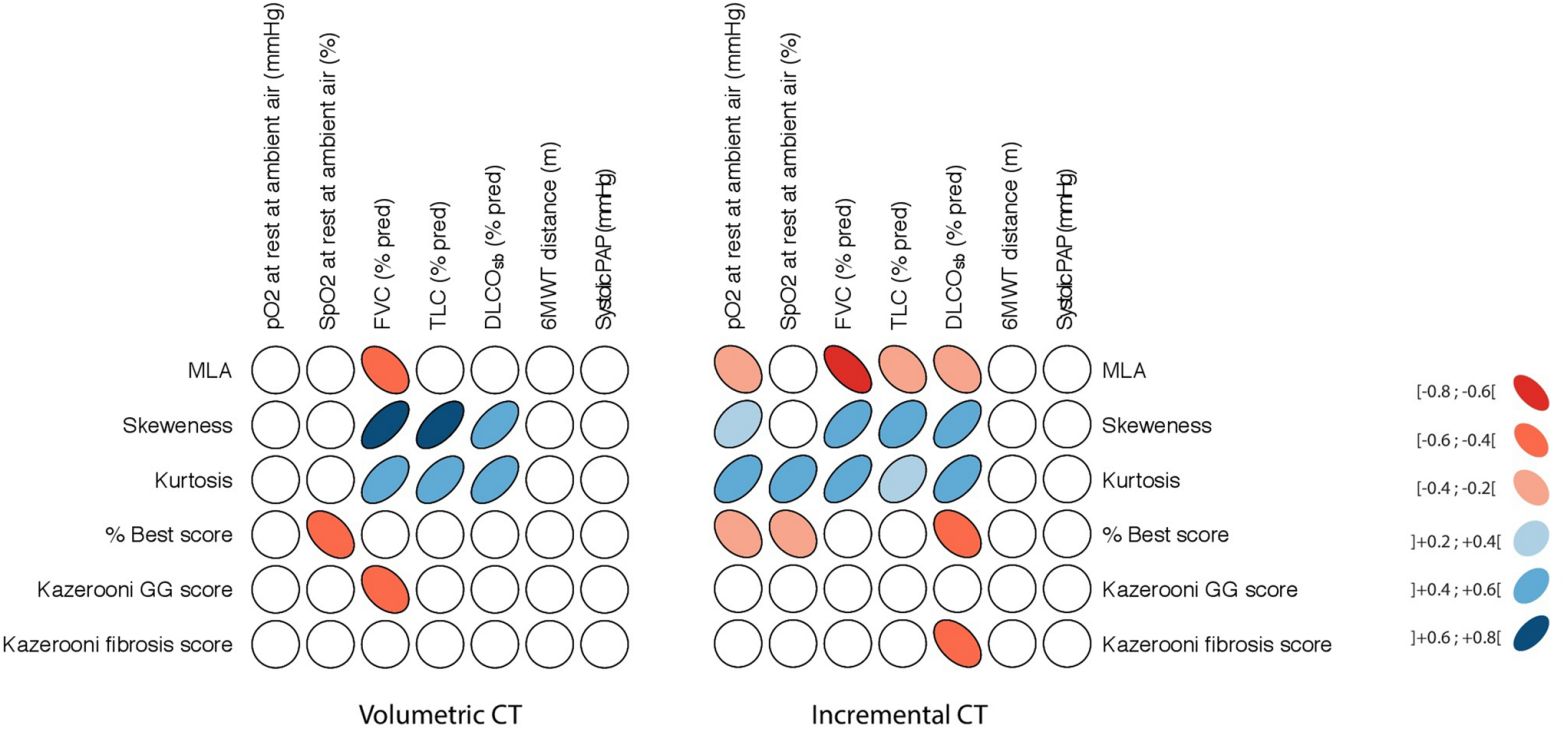
Graphic representation of correlations between density histograms and visual scores with lung function parameters

Correlations between density and visual CT estimates of disease extent and lung function parameters suggestive of disease severity were analyzed for comparison. A graphical representation of the correlation patterns in the two study sub-cohorts is shown in Fig. 2, with analytical results reported in Table 5. The single-breath DLCO was inversely related with the Best (*r* = −0.416; *p* = 0.014), the Kazerooni fibrosis extent (*r* = −0.481; *p* = 0.004) and with the MLA (*r* = −0.382; *p* = 0.026), while a positive correlation was observed with skewness (*r* = 0.583; *p* = 0.001) and kurtosis (*r* = 0.543; *p* = 0.001) in the incremental HRCT study sub-group. Similarly, in the volumetric CT sub-cohort, DLCO_sb_ was significantly correlated only with the density histograms, while the correlation with visual scores was not confirmed. The forced vital capacity (FVC) was exclusively significantly related with all the densitometry indices, with no differences when comparing incremental to volumetric CT. The total lung capacity (TLC) similarly correlated only with density histograms, with similar data in both study sub-cohorts. No correlations were found between the extent of lung changes (visual or quantitative) and exercise performance, as assessed by the distance walked (m) at the six-minute walk test and the estimation of the systolic PAP. Of note, the basal level of arterial oxygen pressure (mmHg) at rest was negatively related to MLA (*r* = −0.331; *p* = 0.037), while a positive correlation was observed with skewness (*r* = 0.379; *p* = 0.023) and kurtosis (*r* = 0.507; *p* = 0.001), in the sole evaluation of incremental HRCT.

**Table 5:** Correlations between density and visual HRCT indices and lung function parameters

		pO_2_ (mmHg)	SpO_2_ (%)	FVC (% pred)	TLC (% pred)	DLCO_sb_ (% pred)	6MWT distance (m)	sPAP (mmHg)
Volumetric CT								
MLA		−0.139 (0.500)	−0.214 (0.294)	−**0.465 (0.019)**	−0.337 (0.158)	−0.315 (0.153)	−0.109 (0.699)	0.039 (0.905)
Skewness		0.29 (0.180)	0.294 (0.174)	**0.656 (0.001)**	**0.612 (0.009)**	**0.581 (0.007)**	−0.158 (0.589)	−0.277 (0.410)
Kurtosis		0.162 (0.483)	0.145 (0.531)	**0.541 (0.011)**	**0.542 (0.037)**	**0.549 (0.018)**	−0.294 (0.329)	−0.129 (0.705)
% Best score		−0.257 (0.206)	−**0.449 (0.021**)	−0.368 (0.071)	−0.286 (0.235)	−0.084 (0.709)	−0.186 (0.506)	0.186 (0.563)
Kazerooni (GG score)		−0.152 (0.458)	−0.27 (0.182)	−**0.413 (0.040)**	−0.284 (0.238)	0.014 (0.950)	−0.448 (0.094)	−0.018 (0.956)
Kazerooni (fibrosis score)		−0.147 (0.474)	−0.171 (0.403)	0.031 (0.881)	0.102 (0.679)	−0.029 (0.897)	0.062 (0.825)	0.034 (0.916)
Incremental CT								
MLA		−**0.331 (0.037)**	−0.248 (0.123)	−**0.616 (<0.001)**	−**0.366 (0.047)**	−**0.382 (0.026)**	−0.178 (0.407)	0.087 (0.681)
Skewness		**0.379 (0.023)**	0.272 (0.108)	**0.561 (0.001)**	**0.436 (0.018)**	**0.583 (0.001)**	0.208 (0.341)	−0.005 (0.984)
Kurtosis		**0.507 (0.001)**	**0.431 (0.007)**	**0.594 (<0.001)**	**0.379 (0.043)**	**0.543 (0.001)**	0.08 (0.71)	−0.355 (0.089)
% Best score		−**0.359 (0.023)**	−**0.318 (0.045)**	−0.136 (0.415)	−0.149 (0.432)	−**0.416 (0.014)**	−0.157 (0.464)	0.176 (0.399)
Kazerooni (GG score)		−0.181 (0.265)	−0.225 (0.162)	−0.058 (0.728)	−0.126 (0.506)	0.077 (0.667)	−0.319 (0.129)	−0.155 (0.461)
Kazerooni (fibrosis score)		−0.137 (0.4)	−0.144 (0.376)	0.063 (0.707)	−0.083 (0.663)	−**0.481 (0.004)**	0.17 (0.426)	0.179 (0.391)

Data are expressed as Spearman correlation coefficient (p values). Statistically significant results (p < 0.05) are reported in bold

Abbreviations: MLA, mean lung attenuation; GG, ground glass; pO_2_, O_2_ arterial partial pressure; SpO_2_, oxygen saturation; FVC, forced vital capacity; TLC, total lung capacity; DLCO_sb_, diffusing lung capacity for carbon monoxide single breath; 6MWT, 6-min walking test; sPAP, systolic pulmonary artery pressure

## Discussion

The present study aimed to investigate the performance of density histograms (MLA, skewness, and kurtosis) along with lung function correlations in a retrospective cohort of IPF patients evaluated by means of incremental or volumetric chest HRCT at the time of first diagnosis. More comfortable to perform, Best and Kazerooni visual scores were evaluated as reference [15, 16]. We found that estimation of both densitometry indices and visual scores did not differ in the two study sub-cohorts whichever was the CT methodology used. This finding was not so expected and likely suggests that differences of lung sampling between the two CT techniques do not influence the overall evaluation of disease extent. Our feeling was further sustained by the fact that patients sub-cohorts were closely comparable in terms of disease severity as assessed by lung function and GAP score. Chest HRCT is the milestone imaging tool for the detection of lung alterations in ILDs, even in the case of diseases at very early stages. Volumetric HRCT has further ameliorated the diagnostic yield of the incremental technique through the tridimensional reconstruction of the entire lung volume that allows the evaluation of additional pathological findings (i.e., neoplasms) and helps the better differentiation of traction bronchiectasis from true honeycombing (notably the average level of agreement between radiologists is less than 50%) [28]. For these reasons, since the beginning of 2015 volumetric has replaced incremental HRCT in our center in the diagnostic workup of patients with suspected fibrotic ILDs. Widely used for monitoring purposes (i.e., disease progression, response to treatment), HRCT, whichever is the technique used, has limited value except if integrated by an appropriate quantification method. Kazerooni et al. [16] first found a good correlation between a visual semi-quantitative scoring system, based on the extent of GGO and fibrosis, and the histology pattern in a cohort of 25 IPF patients. Later on, Best et al. [15] showed that the application of a visual score addressing the percentage distribution of lung abnormalities was predictive of IPF short-term mortality. At the same time, Goh et al. [29] proposed a prognostic algorithm, based on the integration of lung function testing and the CT extent of lung changes, to stratify patients with SS-ILDs. Despite the initial enthusiasm and the broad application of easily to calculate visual scales, the main limitation of their use is represented by the low reproducibility as they are operator dependent [30, 31].

Different computer-based quantification methods have been proposed to ameliorate measurements objectivity, sensitivity, and repeatability. Analysis of density histograms, including MLA, skewness, and kurtosis, has been promisingly applied to IPF and other ILDs. Best et al. [25] initially observed a significant correlation between the FVC and kurtosis in a cohort of 144 IPF patients. Later, a greater extent of lung changes along with lower values of TLC, skewness, and kurtosis was associated with an increased mortality rate [15]. Both studies were retrospective and realized with incremental HRCT. In a prospective study including 48 ILD-SSc cases, Camiciottoli et al. have shown that quantitative indices had a higher reproducibility than visual scoring (incremental CT), and was significantly correlated with lung function and quality of life [11]. Similar observations were reported in 46 patients with biopsy-proven IPF as higher values of skewness and kurtosis along with an increased fibrosis visual score were associated with a shorter transplantation free-survival [32]. Volumetric CT was first used in 2007 by Sverzellati et al. [33] who reported that quantification of lung abnormalities with the fibrotic index (expressed as the ratio between the fibrotic lung volume and the whole lung volume) discriminated IPF patients from controls.

Our study highlights for the first time that both density histograms and visual scores work similarly in IPF patients whichever was the CT technique used (incremental or volumetric). This observation is of clinical relevance as the availability of volumetric CT is not always guaranteed in no ILDs reference centers. This means that patients are not necessarily studied with the same CT while a variable proportion of them may be submitted to both incremental and volumetric CT at different disease stages. Our findings indicate that incremental and volumetric CT may be equally performing for disease extent purposes. The level of concordance between the two radiologists involved in the study was also optimal for both quantitative and visual indices suggesting the high reproducibility of measurements. Of sure, quantitative scoring was a little bit more time consuming than visual analysis. Despite this, the time to master the software was no longer than 1–2 h, making it quite friendly to the user. Main steps of the learning curve were represented by CT scan uploading and ROI setting. The ROI has the task of recognizing the substantial densitometry of the lung tissue thus enabling the software algorithm to discriminate density variations for the calculation of histograms. Overall, this means that the right software management is, in some ways, dependent on the operator expertise which, at least in our opinion, is not necessarily a procedure limit. Undoubtedly, a strength of our approach is that density histograms may be easily computed with an open-source software. Recently, integration of quantitative HRCT with radiomics and lung function has been shown to improve the diagnostic workup of IPF patents [34]. However, efficacy of radiomics is strictly related to the segmentation process, with best results obtained with full automation. Similar considerations can be drawn for additional promising tools like artificial intelligence as the lack of the “critical” point of view of the operator requires further efforts [35, 36].

Certainly, our single-center study is limited because of the retrospective nature and the inability of having a simultaneous intra-patient comparison of the two different CT techniques. A prospective intra-patient study should have been represented the ideal setting that unfortunately is not achievable in clinical practice because of ethical issues due to radiation exposure. Despite this, our patient sub-cohorts were representative of a real-life clinical scenario. Simulating an incremental acquisition from the volumetric images set should also be considered as a choice for intra-patient data analysis. However, in our opinion, it represents an entirely virtual alternative with no application to any clinical setting.

In agreement with previous observations of not spirometry-gated CT scans, quantitative scoring had a better correlation with lung function parameters than visual assessment. Overall, concerning CT technique, we found no significant difference between any pair of correlation coefficients in the whole study population. The presence of a significant flag in one cohort, which was not confirmed in the other one, was only due to the different size consistency of the two samples analyzed. In particular, we confirmed that both FVC and DLCO which are widely accepted as surrogate functional measures of disease severity were correlated with densitometry indices in both incremental and volumetric CT.

The renewed interest in density histograms application is demonstrated by a recent observation showing that a new CT histogram parameter, that is, AROIP (area right of the inflexion point), was predictive of mortality in 70 IPF evaluated by incremental HRCT [37]. We have reported that the integration of density histograms into a composite index-computerized integrated index (CII) could be sufficiently sensitive for capturing early interstitial changes in pulmonary fibrosis related to systemic sclerosis while was predictive of lung function decline [38]. Also, very recently, quantitative CT has been shown to be relevant in the decision-making process of patients with interstitial pneumonia by Sars-Cov-2. Indeed, measurement of lung volume compromise was predictive of oxygen support/intubation and of in-hospital death in a cohort of 222 COVID-19 patients [39].

In conclusion, our study shows that density histograms and visual scores may be applied to incremental and volumetric HRCT to address the disease extent in IPF patients with an expected similar performance. Quantification by density histograms (MLA, skewness, and kurtosis) proves to be superior to visual scoring as more strongly correlated with lung function parameters and displays a preserved optimal reproducibility. Volumetric will soon replace incremental HRCT for ILD assessment in all clinical scenarios, including the estimation of disease progression in clinical trials. At this transition time, we believe that our findings are of clinical interest as represent a sort of bridge toward the near future.

## Data Availability

Data available upon request.
